# Fruit and Vegetable, Fish, and Sugar-Sweetened Beverage Consumption Among Adults Aged 40–65 Years: A Cross-Sectional Study of Dietary Patterns and Sociodemographic Correlates in North-Eastern Poland

**DOI:** 10.3390/nu18182990

**Published:** 2026-09-12

**Authors:** Dominika Jakubowicz-Lachowska, Marcin Bazylewicz, Mateusz Wierciński, Mateusz Bargielski, Emilia Szyłak, Katarzyna Kapica-Topczewska, Anna Mirończuk, Katarzyna Snarska, Monika Chorąży, Alina Kułakowska, Jan Kochanowicz

**Affiliations:** 1 Department of Neurology, Medical University of Bialystok, 15-267 Bialystok, Poland; 2 Department of Clinical Medicine, Medical University of Bialystok, 15-089 Bialystok, Poland

**Keywords:** fruit and vegetable consumption, diet, cardiovascular prevention, cross-sectional study, sociodemographic factors, physical activity

## Abstract

**Background/Objectives**: Fruit and vegetable, fish, and sugar-sweetened beverage (SSB) intake are important modifiable determinants of cardiovascular risk, yet data on these dietary behaviours among middle-aged adults in Poland remain limited. This study assessed their prevalence and sociodemographic correlates. **Methods**: We conducted a cross-sectional study of 4935 adults aged 40–65 years (mean age 54.2 ± 7.0 years; 59.4% women) participating in the “Bet on Prophylaxis—Program for the Prevention of Cerebral Vascular Diseases, in the Masovian, Podlaskie and Warmian-Masurian Voivodeships”. Dietary habits and physical activity (PA) were assessed using a structured questionnaire. Multivariable logistic regression identified independent correlates of each dietary behaviour, including sex × education interactions. **Results**: Daily fruit and vegetable consumption was reported by 67.7% of participants, fish consumption ≥1–2 times/week by 64.1%, and frequent SSB consumption by 14.0%. Daily fruit and vegetable intake was independently associated with female sex (OR = 1.92), higher education (OR = 2.24), older age, and PA (all *p* < 0.001). Women reported healthier fruit and vegetable and SSB habits than men, while fish intake did not differ by sex. Neither body mass index (BMI) nor waist-to-hip ratio (WHR) was independently associated with any dietary behaviour after full adjustment, although WHR showed a weak unadjusted association with poorer fruit and vegetable and SSB habits. A global test of the sex × education interaction was significant for fish consumption (Wald χ^2^ = 15.2, *df* = 3, *p* = 0.002), but not for fruit and vegetable (*p* = 0.622) or SSB consumption (*p* = 0.083). **Conclusions**: Dietary behaviours differed by sex and education, with central adiposity showing a weaker, non-independent association compared with the unadjusted analysis. The association between education and diet differed by sex for fish consumption, being evident mainly among men; a similar pattern for SSB consumption did not reach statistical significance.

## 1. Introduction

Cardiovascular disease (CVD), including cerebrovascular disorders, remains the leading cause of death and disability worldwide, and dietary behaviours are among the most important modifiable risk factors for these conditions. Frequent consumption of SSB has been associated with obesity, type 2 diabetes, and other cardiometabolic disorders, whereas adequate intake of fruit and vegetables and regular fish consumption have been associated with a lower risk of CVD, cancer, and all-cause mortality. However, adherence to dietary recommendations remains insufficient in many populations, including the Polish population [1,2,3]. Improving dietary habits therefore remains a major global public health priority. The World Health Organization (WHO) recommends consuming at least 400 g of fruit and vegetables daily (approximately five servings), regularly including fish as a source of omega-3 fatty acids, and limiting the intake of simple sugars and SSBs as part of CVD prevention strategies [4]. Previous studies have demonstrated that dietary behaviours are influenced by demographic, socioeconomic, and lifestyle factors. Healthier dietary patterns, including higher fruit and vegetable consumption, are consistently observed among women, individuals with higher educational attainment, PA individuals, and non-smokers [1,5].

A recent nationwide Polish study involving nearly 1.2 million participants demonstrated substantial sociodemographic differences in dietary quality. Younger age and lower educational attainment were associated with less favourable dietary patterns, and unhealthy dietary behaviours frequently co-occurred with other risk factors, including physical inactivity and cigarette smoking [6]. Comprehensive data on dietary behaviours in Poland remain limited, particularly among adults aged 40–65 years, a group of particular importance for CVD prevention [7,8]. Poor dietary patterns continue to represent a significant public health challenge in Poland. High consumption of highly processed foods, particularly products rich in added sugars, contributes to the increasing burden of chronic diseases, including obesity, type 2 diabetes, hypertension, and CVD [9]. Despite growing awareness of healthy eating, dietary behaviours and knowledge levels remain strongly influenced by sociodemographic factors, highlighting the need for continued monitoring and targeted preventive interventions [10].

Most previous studies have examined individual dietary habits separately, whereas few have simultaneously assessed fruit and vegetable consumption, fish intake, and SSB consumption within the same well-characterised population. In addition, it remains unclear whether sociodemographic factors, particularly educational level, are associated with these dietary habits in a similar way across different dietary domains. Therefore, the aim of this study was to assess the prevalence of fruit and vegetable, fish, and SSB consumption among adults aged 40–65 years participating in a regional cerebrovascular disease prevention programme in Poland and to identify the sociodemographic and lifestyle factors associated with these dietary habits. A better understanding of these associations may help inform more targeted nutrition education and public health interventions.

## 2. Materials and Methods

A cross-sectional study was conducted as part of the “Bet on Prophylaxis—Program for the Prevention of Cerebral Vascular Diseases, in the Masovian, Podlaskie and Warmian-Masurian Voivodeships”. The program was implemented in 2017–2020. After data verification, 4935 correctly completed questionnaires were available for analysis, including 2932 women and 2003 men; mean age 54.2 ± 7.0 years. The target enrolment of 6000 participants was defined a priori by the programme organisers within the scope of the funding awarded for its implementation; the present study constitutes a secondary analysis of programme data, and no separate sample size calculation was performed. The programme targeted adults aged 40–65 years with no history of a vascular incident (stroke or TIA), i.e., individuals of working age at increased risk of cerebrovascular events, for whom such an event carries substantial occupational and socioeconomic consequences. Participants were recruited in selected primary care clinics, and enrolment was based on self-selection: eligible adults were invited to participate during routine primary care visits. This was therefore not a population-based random sample. Data were collected using a questionnaire specifically designed for this study. The questionnaire included demographic information (age, gender, and education level) and anthropometric measurements, including height, weight (used to calculate BMI), and waist and hip circumference (used to calculate waist-to-hip ratio, WHR). Blood pressure and heart rate were also recorded. Participants were also asked about comorbidities associated with an increased risk of cerebrovascular events, such as hypertension and atrial fibrillation, as well as modifiable lifestyle risk factors, such as smoking, alcohol consumption, physical activity, and dietary habits.

Dietary habits were assessed by asking participants how frequently they consumed fruit and vegetables, fish, and sugar-sweetened beverages (SSBs), using a five-point ordinal scale (never, occasionally, 1–2 times/week, 3–4 times/week, and daily). Participants who reported daily fruit and vegetable consumption were additionally asked to indicate the number of portions consumed per day (1–5). Regular physical activity was recorded as a binary variable (yes/no), and those reporting regular activity also provided its weekly frequency. BMI was calculated from measured height and weight obtained by trained study personnel. Waist-to-hip ratio (WHR), a measure of central (abdominal) adiposity, was calculated from measured waist and hip circumference. The complete questionnaire is available in the Supplementary Material. Continuous variables were expressed as mean ± standard deviation (SD) or median with interquartile range (IQR), depending on data distribution; categorical variables were presented as frequencies and percentages. Comparisons between categorical variables were performed using Pearson’s chi-square test; expected cell counts were verified to exceed 5 in all comparisons, confirming that Fisher’s exact test was not required. Continuous variables were compared between two groups using the independent-samples *t*-test or the Mann–Whitney U test, and across more than two groups using one-way ANOVA or the Kruskal–Wallis test, with non-parametric tests used throughout as a sensitivity check. Correlations between continuous variables were assessed using Pearson’s and Spearman’s rank correlation coefficients.

For the multivariable analyses, the five-level ordinal dietary variables were dichotomised into binary outcomes using the response categories available in the questionnaire. Daily fruit and vegetable consumption was defined as consumption on a daily basis; this reflects the WHO recommendation of daily fruit and vegetable intake [4], although the questionnaire assessed frequency rather than quantity and therefore did not permit verification of the recommended 400 g threshold. Fish consumption was dichotomised at ≥1–2 times per week, the lowest category consistent with regular intake; as the instrument assessed frequency rather than quantity, this variable is reported as “fish consumption ≥1–2 times/week” rather than as adherence to a recommendation. Frequent SSB consumption was defined as ≥3–4 times per week; as no universally accepted frequency threshold exists for SSB consumption, the complementary category (never or occasional consumption) was used as the reference for healthier beverage choices.

The aim of the multivariable models was to estimate independent associations between sociodemographic and lifestyle characteristics and dietary behaviours, rather than to develop predictive models. Covariates included in the primary models were selected a priori on the basis of their established relevance to dietary behaviour in the previous literature and their availability for the entire sample. Variables with a plausibly bidirectional or mediating relationship with dietary behaviour—alcohol consumption frequency, diabetes history, and dyslipidaemia history—were not included in the primary models, as adjustment for these factors could introduce over-adjustment bias; they were instead examined in a separate sensitivity analysis.

Independent factors associated with daily fruit and vegetable consumption, fish consumption ≥1–2 times/week, and infrequent (healthy) SSB consumption were identified using multivariable logistic regression models, with age, sex, education level, BMI, regular PA, smoking status, and hypertension history entered simultaneously. Results were reported as odds ratios (ORs) with 95% confidence intervals (CIs). Sex × education interaction terms were tested for each of the three dietary behaviours to assess whether the association between education and diet differed by sex. The overall significance of the sex × education interaction was assessed using a global Wald test across all interaction terms, with a likelihood-ratio test as confirmation, rather than by interpreting individual interaction coefficients.

Model calibration was evaluated using the Hosmer-Lemeshow goodness-of-fit test, discrimination with the area under the receiver operating characteristic curve (AUC), and multicollinearity using the variance inflation factor (VIF). A Cochran-Armitage test for trend was used to assess linear trends across ordered education categories. As a sensitivity analysis, all three multivariable models were re-estimated with additional adjustment for alcohol consumption frequency, diabetes history, and dyslipidaemia history to assess the robustness of the primary findings to further clinical covariates. In an additional sensitivity analysis, all three models were re-estimated with voivodeship included as a covariate in order to assess the influence of the uneven regional distribution of participants. A two-sided *p*-value < 0.05 was considered statistically significant. All statistical analyses were performed using Statistica 13.1.

## 3. Results

### 3.1. Distribution of Key Continuous Variables

A total of 4935 adults aged 40–65 years were included in the analysis. The mean age was 54.2 ± 7.0 years (median 55, IQR 49–60) and 59.4% were women. Median BMI was 28.3 kg/m^2^ (IQR 25.2–31.8) and median WHR was 0.91 (IQR 0.84–0.97). Given the sample size and the distribution of the continuous variables, both parametric and non-parametric tests were applied, with consistent results.

### 3.2. Dietary Patterns

Table 1 presents the frequency distribution of fruit and vegetable, fish, and SSB consumption. Daily fruit and vegetable consumption was reported by 67.7% of participants, with a mean intake of 2.6 portions per day among daily consumers. Although over two-thirds of participants reported consuming fruit and vegetables daily, only 216 individuals (4.4% of the total sample) reported consuming five portions per day, highlighting a substantial gap between daily fruit and vegetable consumption and adherence to current dietary recommendations. Fish consumption ≥1–2 times/week was reported by 64.1% of participants, whereas frequent SSB consumption (≥3–4 times per week) was reported by 14.0%. Regular physical activity was reported by 33.3% of participants.

### 3.3. Strength of Association: Cramér’s V

For all chi-square tests reported below, expected cell counts exceeded the conventional threshold of five (minimum expected count: 136.9), confirming that the chi-square approximation was appropriate throughout and that Fisher’s exact test was not required. Cramér’s V effect sizes are presented alongside *p*-values in Table 2 to complement statistical significance with an estimate of association strength.

Overall, sex and education showed the strongest associations with daily fruit and vegetable consumption and infrequent SSB consumption, whereas associations with fish intake were consistently weaker. All observed effect sizes were small according to conventional benchmarks.

### 3.4. Sex Differences Across Dietary Behaviours

**Figure 1:**
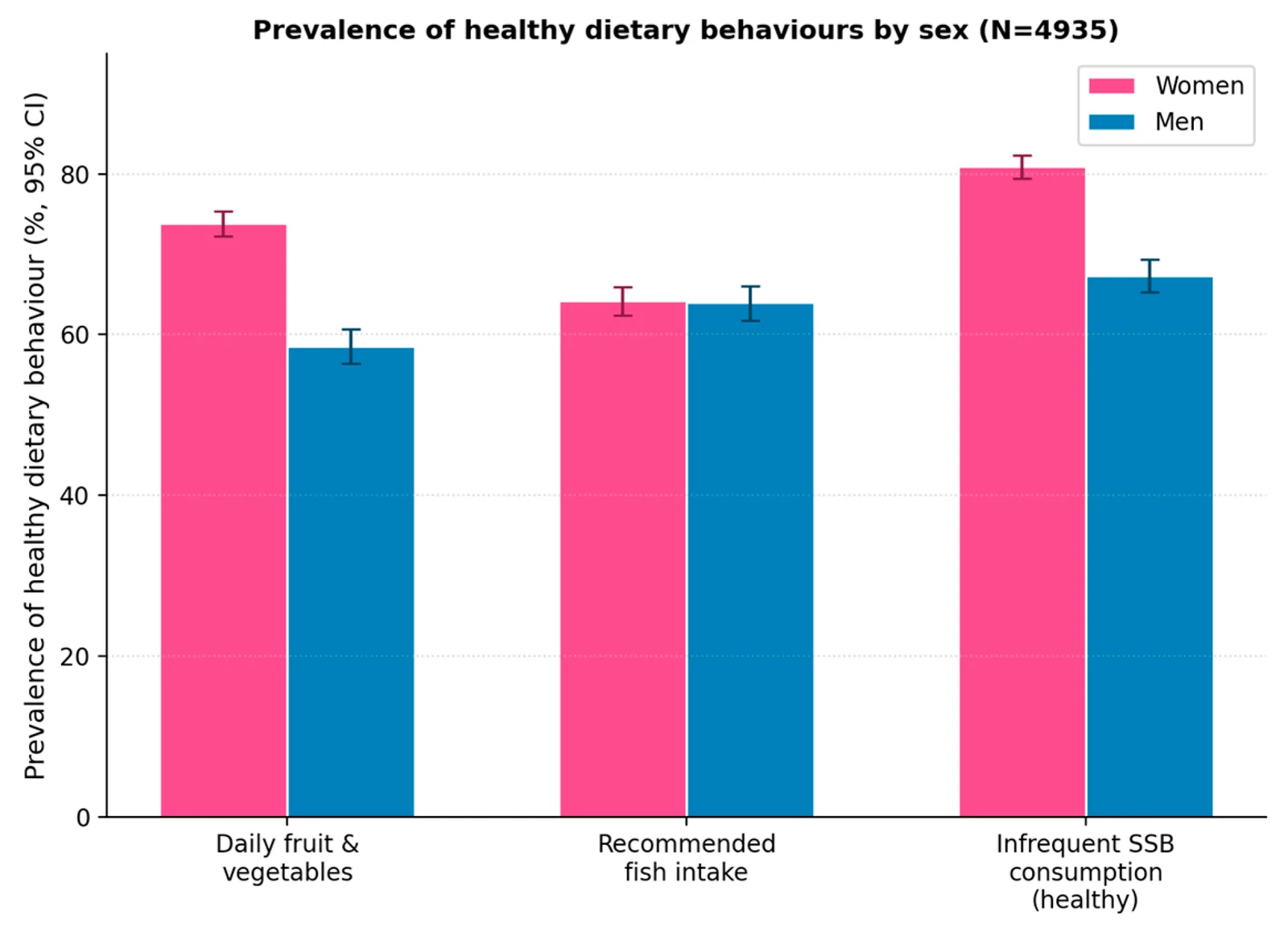
Prevalence of healthy dietary behaviours by sex (*N* = 4935).

Figure 1 shows the prevalence of dietary behaviours according to sex. Women were more likely than men to report daily fruit and vegetable consumption (73.9% vs. 58.6%, χ^2^ *p* < 0.001) and infrequent SSB consumption (81.0% vs. 67.4%, χ^2^ *p* < 0.001). Conversely, frequent SSB consumption was reported by 9.8% of women and 20.2% of men. Fish consumption ≥1–2 times/week did not differ between women and men (64.2% vs. 64.0%, χ^2^ *p* = 0.89).

### 3.5. Adherence to the WHO Recommendation (≥5 Portions/Day)

Adherence to the WHO recommendation of consuming at least five daily portions of fruit and vegetables was analysed separately (Table 3). Only 216 participants (4.4% of the total sample) met this recommendation. In the multivariable logistic regression model, regular PA (OR = 1.62, 95% CI: 1.23–2.14, *p* = 0.001) and non-smoking (OR = 1.52, 95% CI: 1.06–2.19, *p* = 0.023) were independently associated with higher odds of meeting the recommendation, whereas increasing age (OR = 0.98, 95% CI: 0.96–1.00, *p* = 0.046) and higher BMI (OR = 0.97, 95% CI: 0.94–1.00, *p* = 0.033) were associated with lower odds. Female sex (OR = 1.39, *p* = 0.069), waist-to-hip ratio (OR = 0.34, *p* = 0.258), and education level were not significantly associated with adherence to the recommendation. Model performance was modest (Nagelkerke R^2^ = 0.036; AUC = 0.628).

### 3.6. Portion Size Among Daily Consumers

Among participants reporting daily fruit and vegetable consumption, the number of daily portions was weakly positively correlated with education (Spearman’s rho = 0.060, *p* < 0.001) and weakly negatively correlated with WHR (rho = −0.083, *p* < 0.001), whereas no significant correlations were observed with age or BMI.

### 3.7. Multivariable Models for Individual Dietary Behaviours

The three dietary behaviours were only weakly correlated with one another (r = 0.02–0.13), suggesting partial rather than strong co-occurrence among the dietary domains themselves. Table 4 presents the multivariable logistic regression models for each of the three dietary behaviours. All models simultaneously included age, sex, education level, BMI, regular PA, smoking status, and history of hypertension as independent variables. Model performance ranged from modest to acceptable (Nagelkerke R^2^ = 0.081, AUC = 0.647 for fruit and vegetable consumption; R^2^ = 0.017, AUC = 0.566 for fish intake; R^2^ = 0.066, AUC = 0.646 for SSB consumption). Variance inflation factors were below 5 for all correlates (maximum 2.73), indicating no evidence of problematic multicollinearity. The Hosmer–Lemeshow test indicated adequate calibration for the fruit and vegetable and fish models (*p* = 0.098 and *p* = 0.118, respectively), whereas the SSB model showed evidence of poorer calibration (*p* = 0.002). The poor calibration of the SSB model suggests possible model misspecification, such as unmodelled non-linearity or omitted interactions; this is consistent with the non-monotonic BMI–SSB association described below. The odds ratios from this model should therefore be interpreted as indicating the direction of associations rather than as precise effect estimates.

**Figure 2:**
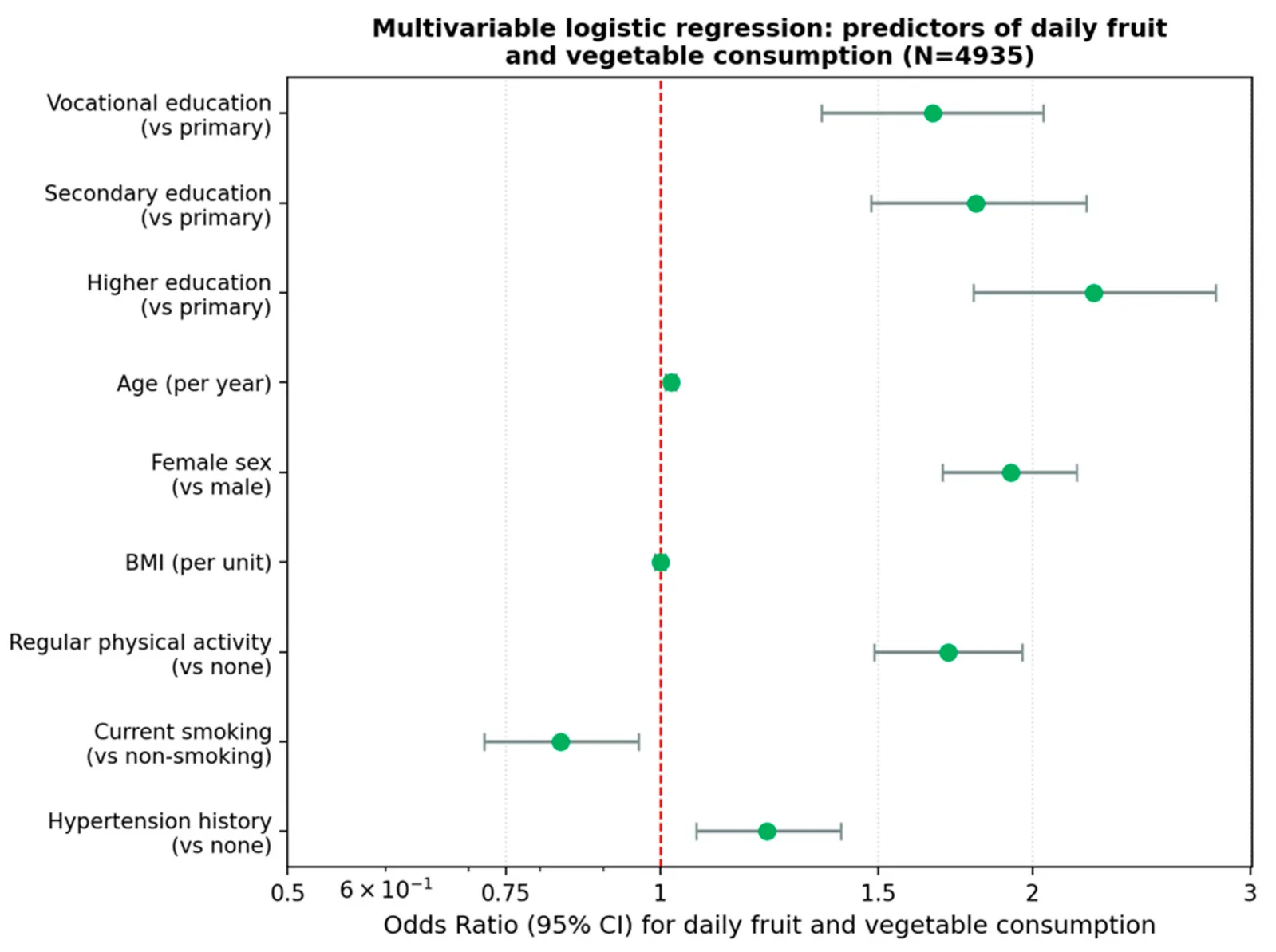
Forest plot of adjusted odds ratios (95% CI) for daily fruit and vegetable consumption (*N* = 4935).

For daily fruit and vegetable consumption (Figure 2, Table 4), the strongest independent correlates were higher education and female sex, with a confirmed positive trend across education categories (Cochran-Armitage *p* < 0.001); BMI was not a significant correlate.

For fish consumption ≥1–2 times/week, education showed no significant linear trend (Cochran–Armitage trend test, *p* = 0.41), consistent with the non-monotonic pattern observed across educational categories. Regular PA and non-smoking remained significant independent correlates. For infrequent SSB consumption, female sex, higher education (Cochran–Armitage trend test, *p* < 0.001), and regular PA were independently associated with higher odds of infrequent SSB consumption, whereas a history of hypertension was associated with lower odds. BMI was not an independent correlate of any of the three dietary behaviours after full adjustment.

### 3.8. Smoking and Dietary Behaviours

Current smokers consistently reported less favourable dietary behaviours than non-smokers across all three dietary measures. Daily fruit and vegetable consumption was reported by 62.2% of smokers compared with 69.5% of non-smokers (*p* < 0.001), fish consumption ≥1–2 times/week by 60.3% versus 65.4% (*p* = 0.0015), and infrequent (healthy) SSB consumption by 70.7% versus 77.1%, respectively (*p* < 0.001). Current smoking also remained independently associated with less favourable dietary behaviours in the multivariable logistic regression models (Table 4).

### 3.9. WHR Versus BMI

In contrast to BMI, WHR was weakly but significantly associated with lower odds of daily fruit and vegetable consumption and infrequent SSB consumption (both *p* < 0.001), whereas no association was observed for fish intake. These findings indicate that WHR was more closely associated with dietary behaviours than BMI in this population. An additional analysis of BMI across the original five-category consumption scales (never to daily) showed that BMI differed significantly across SSB categories (one-way ANOVA *p* = 0.033, Kruskal–Wallis *p* < 0.001), following a non-monotonic rather than linear pattern, whereas the corresponding tests for fruit and vegetable and fish consumption frequency were not significant (both *p* > 0.10). Consistent with this, individuals with obesity (BMI ≥ 30) had significantly higher odds of consuming SSB at least weekly compared with non-obese participants (OR = 1.25, 95% CI 1.09–1.42, *p* = 0.001).

### 3.10. Sensitivity Analysis

As a sensitivity analysis, all three models were re-estimated after additional adjustment for alcohol consumption frequency, history of diabetes, and history of dyslipidaemia. The main findings remained essentially unchanged. Two additional associations were observed: a history of dyslipidaemia was independently associated with higher odds of daily fruit and vegetable consumption, whereas a history of diabetes was independently associated with higher odds of infrequent (healthy) SSB consumption after adjustment for all other covariates. In an additional sensitivity analysis including voivodeship as a covariate, the estimates were essentially unchanged (for daily fruit and vegetable consumption, female sex OR = 1.93 and higher education OR = 2.31, compared with 1.92 and 2.24 in the primary model; differences for all other coefficients were ≤0.03), and none of the substantive conclusions was affected.

### 3.11. Sex-by-Education Interaction Analyses

**Figure 3:**
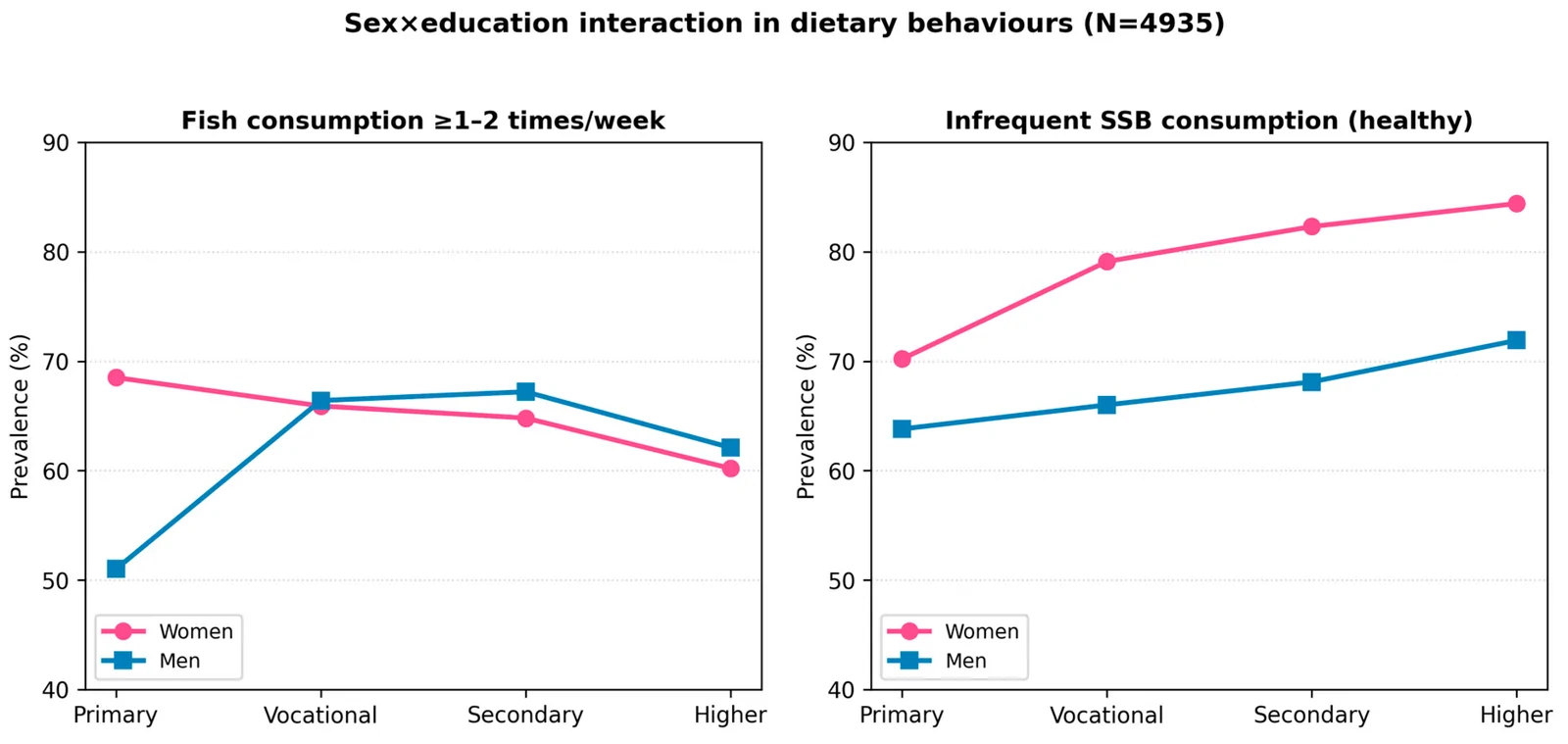
Sex × education interaction for fish consumption ≥1–2 times/week and SSB consumption (*N* = 4935). The global interaction test was statistically significant for fish consumption only.

The overall significance of the sex × education interaction was assessed using global Wald tests. A significant interaction was identified for fish consumption ≥1–2 times/week (Wald χ^2^ = 15.2, *df* = 3, *p* = 0.002), whereas no significant interaction was found for daily fruit and vegetable consumption (χ^2^ = 1.8, *df* = 3, *p* = 0.622) or for infrequent SSB consumption (χ^2^ = 6.7, *df* = 3, *p* = 0.083). Likelihood-ratio tests gave virtually identical results (*p* = 0.621, *p* = 0.002 and *p* = 0.084, respectively). For fish consumption, stratified analyses indicated a positive educational gradient among men that was not apparent among women (Figure 3). For infrequent SSB consumption, stratified analyses were directionally consistent with a steeper educational gradient among women (odds ratios across increasing education categories: 1.76, 2.09 and 2.60 in women versus 1.10, 1.25 and 1.52 in men), although the global interaction test did not reach statistical significance. These findings indicate that the association between education and dietary behaviour differed according to the dietary behaviour examined, and—for fish consumption—also according to sex.

## 4. Discussion

In this large cross-sectional study of adults aged 40–65 years in Poland, adherence to key dietary recommendations varied considerably across food groups and was characterised by pronounced sociodemographic disparities. Although most participants reported daily fruit and vegetable consumption, only a small proportion achieved the WHO-recommended intake of five daily servings. Moreover, clear differences according to sex and educational attainment were observed across all three dietary behaviours examined. Notably, the association between educational attainment and dietary behaviours was not consistent across dietary domains but differed according to both the specific dietary behaviour and sex, a finding that, to our knowledge, has rarely been investigated in such detail.

A particularly notable finding was the substantial discrepancy between the proportion of participants reporting daily fruit and vegetable consumption (67.7%) and the proportion meeting the World Health Organization recommendation of at least five daily servings (4.4%). This adherence rate was markedly lower than the 12% reported for the general European Union adult population in 2019 [11], indicating that adherence to quantitative dietary recommendations in our study population was particularly poor relative to broader European estimates. This pattern is consistent with a recent nationwide Polish study of nearly 1.2 million participants, which similarly reported that younger age and lower educational attainment were associated with poorer overall dietary quality [6]. These results are also consistent with a large Brazilian observational study conducted between 2008 and 2023 (*n* = 697,549), which found that on average 34.1% of adults met the less stringent criterion of regular consumption (≥5 days per week) and 22.5% fully met the recommendation of ≥5 servings per day for ≥5 days per week. In the last years of the study, a decline in both indicators was observed, particularly among those with higher education [12]. These findings indicate that the discrepancy between self-reported daily fruit and vegetable consumption and adherence to dietary recommendations is not limited to Poland or Europe but represents a broader public health challenge. Assessing fruit and vegetable intake using a simple binary measure of daily consumption is likely to overestimate adherence to dietary guidelines because it does not account for the amount consumed. This should be considered when interpreting population-based dietary surveys and developing nutrition education strategies. Our results also highlight the importance of using clear, quantitative public health messages, such as the well-established 5-a-day recommendation, rather than general advice to consume fruit and vegetables “daily” or “regularly”, which may create the impression that dietary recommendations are being met when this is not necessarily the case.

Consistent with previous European studies [1], female sex and higher educational attainment emerged as strong, independent correlates of greater fruit and vegetable consumption, together with older age and regular physical activity. The magnitude of the sex difference observed in our combined fruit and vegetable measure (OR = 1.92, 95% CI 1.69–2.17) fell between the separate estimates reported by Stea et al. [1] for fruit (OR = 1.71) and vegetables (OR = 1.59), which is consistent with our use of a combined measure of fruit and vegetable consumption. Similarly, the education gradient observed in our study (OR = 2.24 for high versus low education) was comparable to the estimates reported by Stea et al. (OR = 1.53 for fruit and OR = 1.86 for vegetables). The same study also reported that participants from Eastern Europe had the lowest odds of consuming fruit and vegetables among all European regions examined [1], which provides relevant context for interpreting our findings in a Polish population. These similarities support the robustness and generalisability of our findings. A Swedish study using an intersectional approach found that inadequate fruit and vegetable intake was most common among men with low educational attainment (81.4%) and least common among women with high educational attainment (47.7%) [13].

WHR showed a weak but statistically significant unadjusted association with lower fruit and vegetable intake, whereas BMI was not an independent correlate of fruit and vegetable consumption in the fully adjusted model. This difference may reflect the distinct characteristics captured by these anthropometric measures, as BMI does not account for body fat distribution, whereas WHR provides information on abdominal adiposity, which may better reflect cardiovascular risk [14]. However, the association between WHR and fruit and vegetable intake was no longer significant after adjustment for sociodemographic and lifestyle factors, suggesting that these factors may explain much of the observed relationship.

Overall, these findings indicate that public health strategies aimed at improving fruit and vegetable intake among middle-aged adults should focus not only on encouraging regular consumption but also on increasing adherence to recommended portion targets, particularly among men and individuals with lower educational attainment.

Unlike the association observed for fruit and vegetable consumption, the relationship between educational attainment and fish consumption differed significantly between men and women, indicating that the effect of education was modified by sex. Fish consumption has well-established clinical importance in CVD prevention.

A large meta-analysis encompassing 40 prospective cohort studies and nearly one million participants demonstrated that higher fish consumption was associated with a lower risk of coronary heart disease (RR = 0.91) and coronary heart disease mortality (RR = 0.85), with each additional 20 g of daily fish intake associated with a 4% reduction in coronary heart disease risk [15]. In our study, 64.1% of participants reported consuming fish at least once or twice weekly. Although this proportion appears relatively favourable, more than one-third of participants consumed fish less frequently.

Several European studies [16,17,18] have reported findings consistent with our results, showing that individuals with healthier overall dietary patterns tend to consume fish more frequently—including higher fruit and vegetable intake among fish consumers in a Dutch cohort [16], greater adherence to a Mediterranean dietary pattern among frequent fish consumers in an Italian population [17], and positive associations with vegetable and fruit intake, physical activity, and non-smoking in a large Swedish cohort [18]. Consistent with our findings, previous research has shown that the association between educational attainment and fish consumption may vary between men and women [19]. In our study, the relationship between educational attainment and fish intake was observed only among men. The very low explanatory power of the fish model (Nagelkerke R^2^ = 0.017) indicates that the sociodemographic and lifestyle characteristics assessed here account for only a small proportion of the variation in fish consumption, suggesting that other factors may be more important determinants of this behaviour. Sex and BMI were not associated with fish consumption, in agreement with the systematic review by Govzman et al., which found no consistent association between these factors and seafood consumption [20]. Although the review examined seafood consumption more broadly, it identified food price, availability, culinary preferences, cooking skills, and health beliefs as important determinants of intake. These findings suggest that fish consumption may be influenced by additional economic, cultural, and behavioural factors beyond the sociodemographic characteristics that are typically associated with other healthy dietary behaviours. We also found that physically active individuals and non-smokers were more likely to consume fish regularly. This is consistent with previous studies showing that fish consumption tends to cluster with other healthy lifestyle behaviours, including higher levels of physical activity and lower smoking prevalence [21]. Additionally, participants with hypertension were more likely to consume fish regularly. This association may reflect greater attention to dietary recommendations among individuals with established cardiometabolic risk factors, as previous studies have shown differences in adherence to nutritional and lifestyle recommendations among patients with cardiometabolic diseases [22]. Previous studies have also shown that fish consumption is often part of a broader healthy lifestyle pattern, being associated with higher levels of physical activity, non-smoking, and higher educational attainment [18].

Furthermore, regular fish consumption is recommended as part of a heart-healthy dietary pattern for cardiovascular risk reduction [21]. Overall, these findings suggest that fish consumption is shaped by a broader and partly different set of determinants than fruit and vegetable consumption, underscoring the need for dietary interventions tailored to specific eating behaviours rather than assuming common correlates across all aspects of diet.

Sugar-sweetened beverage consumption represents one of the most important modifiable dietary risk factors for obesity, type 2 diabetes, and CVD. We therefore examined whether the sociodemographic and lifestyle determinants of this unhealthy dietary behaviour differed from those observed for fruit and vegetable and fish consumption. Our analysis showed that men reported more frequent consumption of SSB than women. This finding is consistent with a global analysis of 185 countries, which identified higher SSB intake among men across populations worldwide [23]. The prevalence of daily and at-least-weekly SSB consumption in our sample (7.9% and 24.5%, respectively) was broadly comparable to, albeit somewhat lower than, that reported in the nationwide Polish study by Zweifler et al. [6].

In our study, we observed that individuals with obesity had a higher odds ratio of SSB consumption (OR = 1.25, 95% CI 1.09–1.42) compared with those without obesity. Similar conclusions were reached in a large Korean national survey, which reported comparable odds ratios for obesity among frequent SSB consumers (OR = 1.38–1.60) [24]. Comparable findings were also reported in a Brazilian population of patients with established atherosclerotic disease, where SSB consumers had significantly higher BMI (*p* = 0.029) and waist circumference (*p* = 0.004) than non-consumers [25]. Building on the BMI–SSB association described above, one methodological observation merits mention: this association was detectable using categorical group-comparison tests but not linear correlation, reflecting a non-monotonic rather than dose–response relationship. This suggests that reliance on linear correlation alone may understate real, non-linear associations between dietary behaviours and anthropometric measures.

Contrary to expectations, a history of hypertension was associated with lower odds of infrequent (healthy) SSB consumption (OR = 0.87, Table 4), indicating more frequent consumption among those with hypertension despite their diagnosis. This may reflect either a lack of sustained behaviour change following diagnosis or reverse causation, whereby higher SSB intake contributes to poorer blood pressure control—a relationship well documented in the opposite direction in prior literature [26]. This is consistent with the nationwide Polish study by Zweifler et al. [6], which similarly found that lower educational attainment was strongly correlated with more frequent consumption of SSB. Our finding of lower SSB consumption among more educated participants is also consistent with a large Estonian study, which reported substantially higher odds of frequent SSB consumption among adults with lower educational attainment (OR = 2.84, 95% CI 1.71–4.74) [27]. This contrasts, however, with a global analysis of 185 countries, which found SSB intake to be higher among more educated adults overall [23], potentially reflecting different stages of nutritional transition across regions.

Taken together, our findings indicate that the relationship between education and dietary behaviour in our study population was not uniform: it was consistent across sexes for fruit and vegetable consumption, whereas for fish consumption it was evident only among men. For SSB consumption, stratified estimates suggested a steeper educational gradient among women, but this interaction did not reach statistical significance and requires confirmation in larger samples. These observations suggest that nutrition education campaigns may need to be tailored to specific dietary behaviours and, at least for some of them, to sex.

The consistent association between current smoking and all three dietary behaviours, together with the relatively weak correlations observed between the dietary behaviours themselves (r = 0.02–0.13), is consistent with previous evidence demonstrating the clustering of unhealthy lifestyle behaviours [28,29]. These findings suggest that the co-occurrence of unhealthy dietary habits and smoking reflects broader patterns of health-related behaviour rather than a single dietary behaviour in isolation. Consequently, lifestyle interventions may benefit from addressing multiple health behaviours simultaneously, including both dietary improvement and smoking cessation.

This study has several important strengths. Its large sample size (*N* = 4935) enabled the identification of independent correlates of dietary behaviours and the formal assessment, using global interaction tests, of whether the association between educational attainment and dietary behaviour differed by sex. By examining fruit and vegetable, fish, and sugar-sweetened beverage consumption within the same population, the study provided a unique opportunity to compare the factors associated with different dietary behaviours and showed that these associations varied across dietary domains. In addition, the analyses included multivariable regression models, interaction analyses, assessment of model assumptions, and sensitivity analyses, supporting the robustness of the findings.

This study has several limitations that should be considered when interpreting the findings. Its cross-sectional design does not permit causal inference, and the observed associations should therefore be interpreted with caution. Dietary behaviours were assessed using self-reported data, which may have been influenced by recall or social desirability bias, leading to some misclassification. Several limitations relate to the dietary assessment instrument. The questionnaire was developed specifically for the prevention programme and was not a validated food frequency questionnaire or dietary recall instrument; no formal validation or reliability testing was performed, which may have introduced measurement error. Furthermore, fruit and vegetables were assessed within a single item, despite the possibility that their determinants and recommended intakes differ; consequently, these food groups could not be analysed separately. Finally, information on portion size was collected only from participants reporting daily fruit and vegetable consumption, which precluded quantitative assessment of intake among less frequent consumers. These limitations should be considered when interpreting the reported adherence estimates, which may be more accurately regarded as indicators of consumption frequency rather than of precise quantitative dietary adequacy. Future studies in this population would benefit from a validated and more granular dietary instrument. In addition, participants were volunteers enrolled in a regional cardiovascular and cerebrovascular disease prevention programme and may not be fully representative of the general population. The study population was also unevenly distributed across the three participating voivodeships, with most participants recruited from the Podlaskie voivodeship. Although a sensitivity analysis including voivodeship did not materially affect the estimates, caution is warranted when generalising the findings to other regions of Poland. Dichotomisation of the ordinal dietary variables, although based on prespecified thresholds, inevitably discards information contained in the original response categories and may render the estimates sensitive to the choice of cut-off. The threshold for SSB consumption is the least firmly grounded in formal guidance, as no established frequency-based recommendation exists for this behaviour. The dietary section of the questionnaire was restricted to fruit and vegetables, fish, and sugar-sweetened beverages. Information on other dietary components relevant to cardiometabolic risk—such as fast food, red and processed meat, or whole grains—and on dietary patterns such as vegetarian or vegan diets was not collected, and these factors could therefore not be taken into account. Participants were recruited from selected primary care clinics; however, clinic identifiers were not available in the dataset provided for this secondary analysis. Consequently, potential clustering of participants within clinics could not be assessed or accounted for using cluster-robust standard errors or multilevel models. As individuals attending the same clinic may share socioeconomic, geographic, and healthcare-related characteristics, the standard errors reported here may be underestimated. As the programme was restricted to adults aged 40–65 years, the findings cannot be extrapolated to younger or older age groups. Finally, the SSB model showed evidence of poor calibration (Hosmer–Lemeshow *p* = 0.002), which may reflect model misspecification, including unmodelled non-linear relationships; estimates from this model should be interpreted with additional caution.

## 5. Conclusions

In this regional Polish population aged 40–65 years, adherence to dietary recommendations differed substantially across dietary domains and sociodemographic groups. Although approximately two-thirds of participants reported daily fruit and vegetable consumption, only 4.4% met the WHO recommendation of at least five servings per day, highlighting the importance of assessing both the frequency and quantity of intake. The correlates of dietary behaviours also differed by food group, with education, smoking, physical activity, and sex showing domain-specific associations, whereas neither BMI nor WHR was independently associated with any of the dietary behaviours examined. These findings support the need for targeted nutrition education and public health strategies tailored to specific dietary behaviours and population subgroups.

## Figures and Tables

**Table 1:** The frequency distribution of fruit and vegetable, fish, and SSB consumption.

*Frequency*	*Fruit&Vegetables, %*	*Fish, %*	*SSB, %*
*Never*	0.6	3.1	36.4
*Occasionally*	3.5	32.8	39.1
*1–2x/week*	8.9	60.2	10.5
*3–4x/week*	19.4	3.5	6.1
*Daily*	67.7	0.3	7.9

**Table 2:** Cramér’s V effect sizes for associations between selected participant characteristics and dietary behaviours.

*Comparison*	*Fruit&Vegetables*	*Fish*	*SSB*
*Sex (Cramér’s V)*	0.160	0.002	0.155
*Education (Cramér’s V)*	0.124	0.052	0.101

**Table 3:** Multivariable logistic regression for adherence to the WHO recommendation of ≥5 daily fruit/vegetable portions (216 events, *N* = 4935).

Predictor	OR	95%CI	*p*
**Age (per year)**	0.98	0.96–1.00	0.046
**Female sex (vs** **.** **male)**	1.39	0.98–1.98	0.069
**BMI (per unit)**	0.97	0.94–1.00	0.033
**WHR**	0.34	0.05–2.19	0.258
**Regular physical activity**	1.62	1.23–2.14	0.001
**Current smoking**	0.66	0.46–0.94	0.023
**Vocational education (vs** **.** **primary)**	0.72	0.43–1.22	0.223
**Secondary education (vs** **.** **primary)**	1.10	0.68–1.79	0.684
**Higher education (vs** **.** **primary)**	0.87	0.52–1.47	0.610

**Table 4:** Multivariable logistic regression models (odds ratios) for daily fruit/vegetable consumption, fish consumption ≥1–2 times/week, and infrequent (healthy) SSB consumption, each adjusted for age, sex, education, BMI, PA, smoking, and hypertension history (*N* = 4935).

Predictor.	Fruit & Veg. Daily, OR	Fish, OR	SSB Rare (Healthy), OR
Age (per year)	1.02	1.01	1.04
Female sex (vs. male)	1.92	1.01	1.89
Vocational education (vs. primary)	1.66	1.28	1.36
Secondary education (vs. primary)	1.80	1.24	1.59
Higher education (vs. primary)	2.24	1.00	1.98
BMI (per unit)	1.00	1.00	0.99
Regular physical activity (vs. none)	1.71	1.27	1.21
Current smoking (vs. non-smoking)	0.83	0.80	0.81
Hypertension history (vs. none)	1.22	1.17	0.87

## Data Availability

The data presented in this study are available on request from the corresponding author due to privacy restrictions.

## Supplementary Materials

The following supporting information can be downloaded at: https://www.mdpi.com/article/10.3390/nu18182990/s1, File S1: Clinical Assessment Checklist (English translation of the questionnaire used in the “Bet on Prophylaxis—Program for the Prevention of Cerebral Vascular Diseases”).
