# Supplementary material for: Fruit and Vegetable, Fish, and Sugar-Sweetened Beverage Consumption Among Adults Aged 40–65 Years: A Cross-Sectional Study of Dietary Patterns and Sociodemographic Correlates in North-Eastern Poland
Source: Nutrients. 2026 Sep 12;18(18):2990. doi: 10.3390/nu18182990 (PMC13610717; doi:10.3390/nu18182990)
Supplement: Supplementary file 1 [file nutrients-18-02990-s001.zip › nutrients-4519475-supplementary.pdf]

SUPPLEMENTARY MATERIAL

CLINICAL ASSESSMENT CHECKLIST

Developed for the "Bet on Prophylaxis – Cerebrovascular Disease Prevention Program"

Original language: Polish. English translation provided for publication purposes.

Name: ____

Date of completion: ________________________________

Personal identification number (PESEL): _____________

Education:

☐ Primary ☐ Vocational ☐ Secondary ☐ Higher

Please indicate the nature of your work:

☐ Mental/office work ☐ Physical work ☐ Not professionally active

ANTHROPOMETRIC MEASUREMENTS

1. Body weight: _____________

2. Height: _____________

3. Waist circumference: _____________

4. Hip circumference: _____________

CARDIOVASCULAR ASSESSMENT

5. Arterial hypertension: ☐ Yes ☐ No

6. Blood pressure, right arm: _____________

7. Blood pressure, left arm: _____________

8. Heart rate: _____________

9. Regular heart rhythm: ☐ Yes ☐ No

10. Carotid artery bruit: ☐ Yes ☐ No

MEDICAL HISTORY

11. History of diabetes treated with oral medications: ☐ Yes ☐ No

12. History of diabetes treated with insulin: ☐ Yes ☐ No

13. Carbohydrate metabolism disorders – adherence to a diabetic diet: ☐ Yes ☐ No

14. Lipid metabolism disorders – adherence to a low-fat diet: ☐ Yes ☐ No

15. History of atrial fibrillation: ☐ Yes ☐ No

16. Do you experience palpitations or an irregular heartbeat? ☐ Yes ☐ No

TOBACCO USE

17. Do you smoke tobacco products (cigarettes, pipe, cigars)? ☐ Yes ☐ No

18. How many cigarettes do you smoke per day?

☐ 1–5 ☐ 6–10 ☐ 11–15 ☐ more than 16

19. If you do not currently smoke, have you ever smoked? ☐ Yes ☐ No

20. If yes, how many years ago did you stop smoking? (please specify the year): _____________

21. Is tobacco smoked in your presence at home? ☐ Yes ☐ No

22. Do you use electronic cigarettes (e-cigarettes)?

☐ Yes – strength/type: _____________ ☐ No

23. Have you used any pharmacological aids for smoking cessation? (please specify: tablets, chewing gum, spray, etc.)

____

ALCOHOL CONSUMPTION

24. Do you consume alcohol? ☐ Yes ☐ No

25. How often do you consume alcoholic beverages?

☐ Daily ☐ 3–4 times/week ☐ 1–2 times/week ☐ Occasionally

26. If you consume alcohol, what type and what volume do you usually consume?

____

DIETARY HABITS

27. How often do you eat fish?

☐ Daily ☐ 3–4 times/week ☐ 1–2 times/week ☐ Occasionally ☐ Never

28. How often do you drink sugar-sweetened carbonated beverages?

☐ Daily ☐ 3–4 times/week ☐ 1–2 times/week ☐ Occasionally ☐ Never

29. How often do you eat fruit and vegetables?

☐ Daily ☐ 3–4 times/week ☐ 1–2 times/week ☐ Occasionally ☐ Never

30. If you eat fruit and vegetables daily, how many portions per day?

☐ 5 ☐ 4 ☐ 3 ☐ 2 ☐ 1

PHYSICAL ACTIVITY

31. Do you engage in regular physical activity? ☐ Yes ☐ No

32. If you engage in regular physical activity, how many times per week?

☐ 1× ☐ 2× ☐ At least 3× ☐ 4× ☐ At least 5× ☐ 6× ☐ Daily

PSYCHOACTIVE SUBSTANCES

33. Do you use psychoactive substances? ☐ Yes ☐ No

NEUROLOGICAL SYMPTOMS (LIFETIME HISTORY)

34. Have you ever experienced dizziness? ☐ Yes ☐ No

35. Have you ever experienced sudden loss of vision in one eye? ☐ Yes ☐ No

36. Have you ever had your visual field obscured on one side? ☐ Yes ☐ No

37. Have you ever experienced weakness of an arm and/or leg? ☐ Yes ☐ No

38. Have you ever experienced speech disturbances or difficulty understanding speech? ☐ Yes ☐ No

39. Have you ever experienced facial drooping? ☐ Yes ☐ No

40. Have you ever experienced sensory disturbances? ☐ Yes ☐ No

41. Have you ever experienced a sudden, very severe headache? ☐ Yes ☐ No

FAMILY AND RECENT MEDICAL HISTORY

42. Has anyone in your family had a stroke? If yes, who?

☐ Yes: _____________ ☐ No

43. Has this person, within the last month, experienced a transient ischaemic attack (TIA), i.e. an episode of focal neurological deficit limited to a specific area of the brain or retina, caused by ischaemia within one vascular territory of the central nervous system, lasting no longer than 24 hours, manifesting as: speech disturbances (aphasia), paresis, sensory disturbances, or transient monocular blindness (amaurosis fugax)?

☐ Yes: _____________ ☐ No

44. In connection with the situation described in question 43, was this person investigated? If yes, when and what was the diagnosis?

☐ Yes: _____________ ☐ No
